# Investigating Health Context Using a Spatial Data Analytical Tool: Development of a Geospatial Big Data Ecosystem

**DOI:** 10.2196/35073

**Published:** 2022-04-06

**Authors:** Timothy Haithcoat, Danlu Liu, Tiffany Young, Chi-Ren Shyu

**Affiliations:** 1 Institute for Data Science and Informatics University of Missouri Columbia, MO United States

**Keywords:** context, Geographic Information System, big data, equity, population health, public health, digital health, eHealth, location, geospatial, data analytics, analytical framework, medical informatics, research knowledgebase

## Abstract

**Background:**

Enabling the use of spatial context is vital to understanding today’s digital health problems. Any given location is associated with many different contexts. The strategic transformation of population health, epidemiology, and eHealth studies requires vast amounts of integrated digital data. Needed is a novel analytical framework designed to leverage location to create new contextual knowledge. The Geospatial Analytical Research Knowledgebase (GeoARK), a web-based research resource has robust, locationally integrated, social, environmental, and infrastructural information to address today’s complex questions, investigate context, and spatially enable health investigations. GeoARK is different from other Geographic Information System (GIS) resources in that it has taken the layered world of the GIS and flattened it into a big data table that ties all the data and information together using location and developing its context.

**Objective:**

It is paramount to build a robust spatial data analytics framework that integrates social, environmental, and infrastructural knowledge to empower health researchers’ use of geospatial context to timely answer population health issues. The goal is twofold in that it embodies an innovative technological approach and serves to ease the educational burden for health researchers to think spatially about their problems.

**Methods:**

A unique analytical tool using location as the key was developed. It allows integration across source, geography, and time to create a geospatial big table with over 162 million individual locations (X-Y points that serve as rows) and 5549 attributes (represented as columns). The concept of context (adjacency, proximity, distance, etc) is quantified through geoanalytics and captured as new distance, density, or neighbor attributes within the system. Development of geospatial analytics permits contextual extraction and investigator-initiated eHealth and mobile health (mHealth) analysis across multiple attributes.

**Results:**

We built a unique geospatial big data ecosystem called GeoARK. Analytics on this big table occur across resolution groups, sources, and geographies for extraction and analysis of information to gain new insights. Case studies, including telehealth assessment in North Carolina, national income inequality and health outcome disparity, and a Missouri COVID-19 risk assessment, demonstrate the capability to support robust and efficient geospatial understanding of a wide spectrum of population health questions.

**Conclusions:**

This research identified, compiled, transformed, standardized, and integrated multifaceted data required to better understand the context of health events within a large location-enabled database. The GeoARK system empowers health professionals to engage more complex research where the synergisms of health and geospatial information will be robustly studied beyond what could be accomplished today. No longer is the need to know how to perform geospatial processing an impediment to the health researcher, but rather the development of how to think spatially becomes the greater challenge.

## Introduction

Health researchers need integrated social, environmental, and infrastructural information to extend the scope of health care and address the complex questions and contextual relationships surrounding health outcomes. Any given location is associated with different contexts—physical, biological, environmental, infrastructural, economic, social, and cultural—all of which can affect population health, disease risk, and access to health care. Geographic context plays a growing role in connecting heterogeneous geoenabled information, especially in health research [1-4]. Spatial context includes elements and interactions with both the societal and the physical infrastructures associated with an individual’s daily activities. This includes accessibility, surrounding natural and built environments, social behaviors, and any related location-specific exposures, understanding that these elements change across geographic areas, scales, and time. Impactful health research that can be applied to real-world issues and problems must be grounded within the context of place [5-7]. Location and the location’s context both matter [8-10]!

The strategic transformation of population health, epidemiology, and eHealth studies require vast amounts of integrated digital data to create understanding that can then support decisions [11]. Questions asked today are more complex than ever before, implicitly tied to understanding context [12-18]. Health researchers have used the Geographic Information System (GIS) to identify, mitigate, and address a myriad of factors affecting health disparities [19-22], health assessments [23-26], health-environment interactions [27-32], health-cultural interactions [33-35], and health service access [36-42]. GIS analysis is expanding within health analysis, but its use is often focused on thematic single-variable maps and their visualization [43-45]. Medical researchers who study health disparities tend to focus on demographic, social, or economic variables from local to national levels, both cross-sectional and over time, that are available from the decennial census or the American Community Survey (ACS). Although there are exceptions [46], far fewer use variables related to the natural, physical, or built environment, primarily because they are more challenging to obtain.

Although advancement is evident in the various web-mapping sites across the federal health realm (the Centers for Disease Control [CDC] and Prevention’s Heart Disease and Stroke Maps, the National Institutes of Health [NIH] and National Cancer Institute’s Cancer Atlas and state profiles, and the Environmental Protection Agency’s [EPA] EnviroAtlas), several issues persist. Although integrated information sources available for researchers are growing [47-49], they each portray only a specific view of that entity’s mandated purview. Most provide visualization of singular attributes at a time and rely on the user to mentally synthesize these pieces of information to generate understanding. It remains a challenge for health researchers to locate and evaluate what specific attributes exist and at what geographies. Moreover, many health researchers “don’t know what they don’t know” with regard to geospatial data. The ability to create new hypotheses is missed if researchers are not aware of the availability of data or the types of questions that could be posed that could further expand their research. More importantly, development of contextual relationships among variables could be discovered through spatial analytics. In addition, the quantification of interactions of health, demographics, infrastructure, and environmental elements in terms of interplay and synergy remains elusive.

Needed is a novel analytical framework designed to leverage location to create new contextual knowledge and associations among otherwise disjointed data. This would aid evidence-based exploration of relationships among layers, discover patterns of interaction, and support clinical sampling designs where quantification and location are interwoven. This paper outlines a new big data approach to building and evolving such a geoenabled health information system. The Geospatial Analytical Research Knowledgebase (GeoARK) is an informatics and data science solution that uses advanced complex contextual queries across multiresolution locational information to geoenable health research.

The objective of GeoARK is to transform attitudes and empower health research where real-world problems are examined in geoenabled context. We can gain efficiencies through integrated heterogeneous public information sources and the establishment of context through geospatial measures, such as proximity, adjacency, network analysis, and spatial analysis. These then form a new complex of attributes within a single geoenabled knowledgebase. It can support a broad spectrum of health research, including health disparities, telemedicine, communicable disease management, zoonotic disease surveillance, environmental health, and health access policy making. It enables eHealth researchers to bring their own collection of eHealth or mobile health (mHealth) events and have user-selected attribute data compiled at those points or output artificial intelligence/machine learning (AI/ML)-friendly databases for further analysis. The contextualization of existing research would enhance the scope of that research.

## Methods

### GeoARK Design

**Figure 1:**
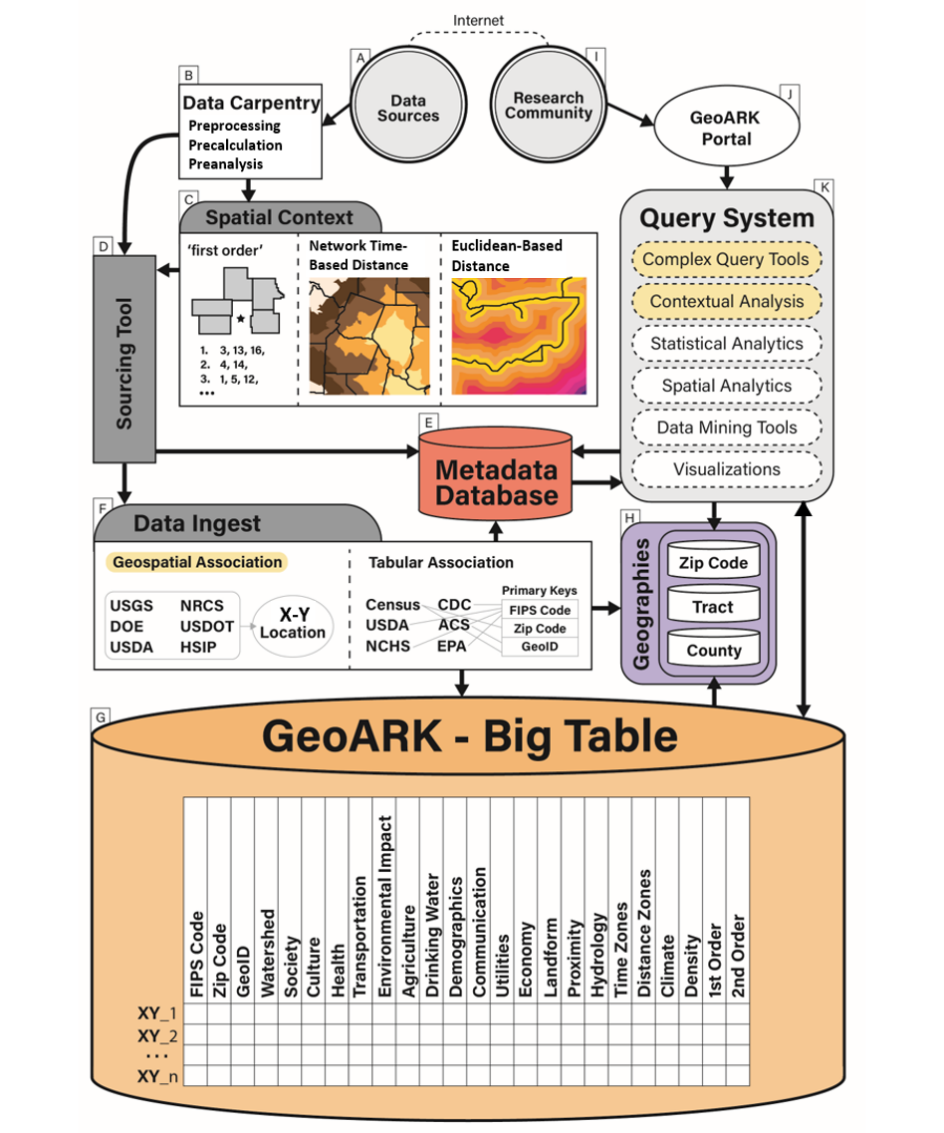
Geospatial Analytical Research Knowledgebase (GeoARK) System design.

This paper describes GeoARK and its potential to greatly extend eHealth research. It outlines how the system was designed and demonstrates how its design leads to its potential within health research. The GeoARK system (Figure 1) comprises multiple components that interact to form a complete process for the integration, documentation, and spatial registration of data into a single queryable big table that we call GeoARK-Big Table (GeoARK-BT) in this paper. It can be used by health researchers to accelerate the use of spatial data and exploit local context within analyses.

The actual spatial framework of GeoARK-BT is based on a dense distribution of points across the United States. The spatial base is a hexagon tessellation of points blanketing the United States at a spacing of 161 m (1/10th of a mile, or 528 ft). Centroids of census blocks with an area less than 67,261 m^2^ (16.6 acres) are integrated into the tessellation to better capture features in more densely populated areas. Proximal polygons are calculated for each point that allows for area totals as well as aggregation into user-specified geographies to occur. These units form a coherent framework for cataloging data over geographical space. The point locations create the sampling framework through which GeoARK captures and encodes the locational variability that exists across the databases integrated. For the United States, there are 162 million points with basic information (5549 attributes) in our current system. Each point is a row, with all attributes associated with that location becoming columns in the database, while each attribute is a column with 162 million rows, with each element of the column representing a specific location’s attribute. The points are stored in a Hadoop Distributed File System (HDFS). The total size is 12.5 TB. The data loading process for all 5549 attributes across the 50 states took 2585 min using a Dell PowerEdge R740xdcompute node with a dual Intel(R) Xeon(R) Gold 6138 CPU (80 cores) and 384 GB memory.

### Data Sourcing and Metadata

The GeoARK system integrates interdisciplinary public data existing in a wide variety of formats (tabular, raster, point, line, and polygon). This increases the efficiency of research since many data elements and sources are challenging for researchers to compile and uniformly integrate for analysis. The GeoARK-BT includes demographic, social, economic, educational, cultural, infrastructure, and environmental attributes from a growing variety of sources, as listed in Multimedia Appendix 1.

A sourcing tool was developed to standardize the collection, documentation, and logging of each data source being added into the GeoARK-BT collection to ensure data quality and integrity for long-term tracking and maintenance. Once a data source is identified, it is added to the GeoARK source table and then data set information is collected and compiled into the data set descriptive listing (ie, data use agreements, constraints, URLs). The metadata database then catalogs and records individual attribute information from these data sets. The metadata database includes sources, metadata (for both data sets and their associated attributes), and attribute links for the GeoARK-BT. The NIH’s Findable, Accessible, Interoperable, Reusable (FAIR) initiative [50] provides a use area for this metadata. Data added to the big table use the attribute lookup table to set attribute field names. Data sources and attribute fields have also been assigned to an International Organization for Standardization (ISO) 19115 thematic category [51]. Natural language tags describing each attribute were also added. Once attributes are loaded, these metadata elements facilitate discovery, query, crediting, and reuse, with all metadata fields being searchable using MongoDB Query Language. Once attribute selection is performed by the researcher, and a data extract is created, a report summarizing the data source information for all data elements contained in the selection is generated. This facilitates the methodological aspects of data collection and documentation for researchers.

### Data Ingestion

Relevant open data sources are ingested to the GeoARK system as tabular information or as relative geographic locations. Although these data independently have great singular value, combining these data, using location as the linkage between data sets, is the power of geospatial analysis and the underpinning for the GeoARK system. Data carpentry and preprocessing are required for some data sources and elements. Attributes being used as links need to be standardized, and categorical data need to be transformed. In some cases, new derived attributes are calculated through aggregation of existing attributes. Precalculations of percentages, densities, means, quantile breaks, and the results of spatial-based analyses further extend the database. By transforming the raw numeric counts into density measures (ie, population, race, ethnicity, or other density per km^2^), we can then tally the points and their areas that are within the area of interest or meet a selection criterion, and derive estimated values for these attributes. This can be accomplished without the need for standard spatial layer intersection procedures where calculation of crossing vectors is required. The process is simply a point in polygon selection. This process allows GeoARK great flexibility in context quantification for applied digital health research.

Tabular data linkage was obtained by a common attribute. Each GeoARK-BT point has been identified as being within a specific Census 2010 block, Census 2020 block, 5-digit zip code, and specific watershed code. Any data sharing a common key could then be added. Data collected at a native geographic level, such as county, zip code, or tract, are loaded directly. For information cataloged at finer units such as block groups and blocks, the associated data are loaded into the GeoARK-BT using the appropriate census link for each point. Scripts for ingestion to, or update of, the GeoARK-BT for recurring data sources (ie, ACS updates) include extract, transform, and load processes for these sources. The data are synced with the GeoARK system to add new, updated, or changed elements.

For geospatial data, linkage was obtained by the X-Y location. Line-based spatial data, such as road networks, and point-based data, such as hospitals, nursing homes, and public health clinics, have been integrated within the GeoARK-BT. To do so, these files needed processing so as to align with the GeoARK points. Data that were spatially analyzed for contextual measures (buffers, Euclidean distance, network time, etc) were converted into polygon form or a raster representation. These layers were then associated with each GeoARK-BT point and the travel time or distance for the feature assigned. Some data may be categorical (ie, land cover or soils) or continuous in nature (ie, elevation or precipitation), further effecting ease of integration. Such files were directly assessed against the GeoARK-BT proximal polygon representation to generate a series of attributes that capture the values’ variability at that location for these data types.

### Context Measures

An innovative aspect of GeoARK is that it has precalculated spatial context measures for many features. The simplest contextual measure is presence within a geography or gridded cell. In another form, context is represented as proximity between a location and features of interest (ie, distance from the stroke unit). It can also take the form of a distance from a linear object (ie, power lines). Proximity can also be derived from network modeling to obtain measures of remoteness, isolation, and accessibility (ie, time or distance).

Density measures utilize a grid or distance to tally the number of points, total length of lines, etc, to generate per area metrics. Data such as block-level population, transmission lines, railroads, confined animal feeding operations, and drinking water wells would be cataloged into artificial grids for this density mapping.

Context is also quantified by identifying first- and second-order spatial relationships within geographic levels. These can be thought of as adjacent neighbors and are identified using spatial analytics. For a given county, the first order is all counties adjacent to this base county. The second order for that same county is all the counties that are adjacent to the first-order counties. These attributes quantify geospatial adjacencies that health researchers can exploit.

**Figure 2:**
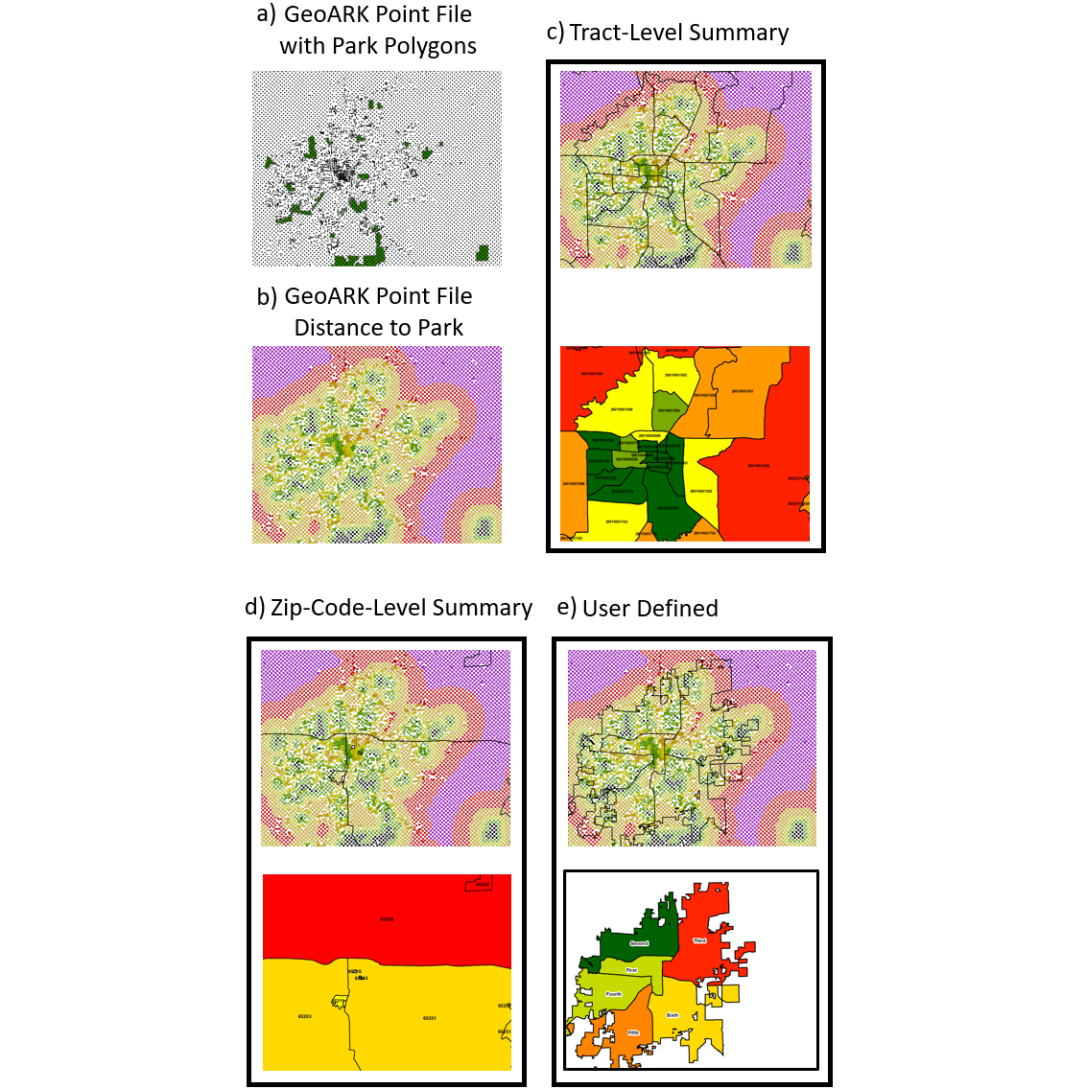
This example shows how GeoARK point processing based on a single attribute (Distance to Parks) can be used to generate summaries at various geographic levels. a) Shows GeoARK point layer with parks data superimposed. b) Shows GeoARK points colorized to show distance from parks inherent in their attribution. c-e) Show dark outlines of tract, zip code, and user defined interest - voting wards (respectively) superimposed on the colorized GeoARK points and below each is their resulting geographic summary for mean Distance to Parks. GeoARK: Geospatial Analytical Research Knowledgebase.

Finally, geographic summary levels that are commonly used in research and mapping are precompiled. Although many attributes can be directly related using relational joins at a specific geographic level (county, tract, zip code), other attributes such as distance measures, land cover, elevation, and climate need to be aggregated from the GeoARK-BT points to generate a summary attribute (ie, mean distance to parks) from these features for any region (Figure 2).

### GeoARK Utility

The collection, integration, and use of diverse data are foundational to answer today’s health problems. Significant disparities exist and can vary across scales from blocks to neighborhoods to regions [3]. In addition, a complex myriad of factors that can affect disparities also exists [52]. In rural contexts [19,36], aging populations, health care access [12,13], sparse populations, environmental exposures [14,15,28], and infrastructure [20] are proven critical factors. In urban contexts, food-deserts [21], crime density and stress [53], and pollution (air, water, light, and noise) [16,54] play possible roles. How do these factors interact? At what scale are these associations important? Where are these findings located, and are they clustered?

The proposed web portal will provide tools to enable and catalyze a health researcher’s ability to move their question into the spatial realm and analyze their area of interest against the broad spectrum of data within the GeoARK-BT. An investigator’s area of interest could be an actual physical area (ie, neighborhood, zip code, place) or a collection of health events as X-Y coordinate pairs with which to associate GeoARK attributes. Complex queries can be used to create and refine data extracts that focus on a researcher’s question of interest.

To support research, flexible access and powerful interrogation of the GeoARK-BT are required. One of the major strengths of GeoARK is the streamlining of access across data sources and the provision of complex analytic query across multiple timestamps and sources. The query is simply a projection on selected columns within the GeoARK-BT using MongoDB. Indexes were built off-line on each attribute to allow for more efficient on-demand retrieval of information. A single-attribute index takes 623 min to build, and a composite index with 5 attributes takes 791 min. Each index, respectively, has, on average, a 0.66 and 1.05 GB memory footprint for a single and a composite index. Open source analytical tools are to be added to provide further analytical functionality to include descriptive, exploratory, inferential, causal, and predictive approaches to targeted spatial analytical research as GeoARK matures. Points can be selected based on user-defined areas of interest and then aggregated to create a surrogate representation of that area and used to extract user-selected attributes from GeoARK to create a subset for further analysis. The design leverages a big data table where we can have high throughput for data transactions.

The 7 query types, listed in Table 1, range from simple attribute selection to queries that utilize the distance to or from a specific feature type to those that require network travel time or distance. Others might include multitemporal queries concerning what has changed since a particular event or point in time. Still others inquire about features and elements around a particular place or location and the associations found between those factors. Finally, other queries can be built to determine or assess how scale or geographic extent may impact conclusions. Output from each of these types of queries can produce AI/ML-ready data sets leveraging GeoARK’s spatial bins and analytical associations.

There is no equivalent system currently available with which to provide side-by-side analytics. When the times presented are compared to the time savings a researcher would obtain through the system’s integrated and spatially contextualized information, they provide great value. In addition, through further testing of indexing schemas and optimization of query and search designs, these times are expected to decrease.

**Table 1:** Examples of query types and their run times when executed against the national GeoARK^a^ database. These can range from national to local studies. The first 3 query examples are standard selections based on attribute values or thresholds. The next 3 query examples illustrate the use of the unique spatial dimensional attributes added through the GeoARK system to provide greater geoanalytical power to selections. The final example demonstrates GeoARK’s ability to select contextual elements that surround another feature of interest.

Query type	Query example	Query time (min)
Simple geography	Select all records for the state of Missouri, Federal Information Processing Standard (FIPS) code=29.	13.03
Simple variable	Select all county records with a nonmetro flag (2013)=1 in Missouri.	5.81
Complex variable	Black/African American % of total population of zip code >30% AND % total population in poverty >15% AND % households with a single female head of household with children under 18 years of age receiving food stamps >5%.	5.47
Density	Select points with a road density greater than 1500 m (4921 ft) per square kilometer.	0.17
Proximity	Select points with a distance to closest park greater than 400 m (1312 ft).	0.14
Travel time	Select points having 15 min or less travel time to the nearest hospital.	0.07
Contextual	Given a cluster of 3 counties with high cancer incidence, compile and extract all surrounding counties’ exposome variables associated with those locations.	1.23

^a^GeoARK: Geospatial Analytical Research Knowledgebase.

## Results

### Case Studies

**Figure 3:**
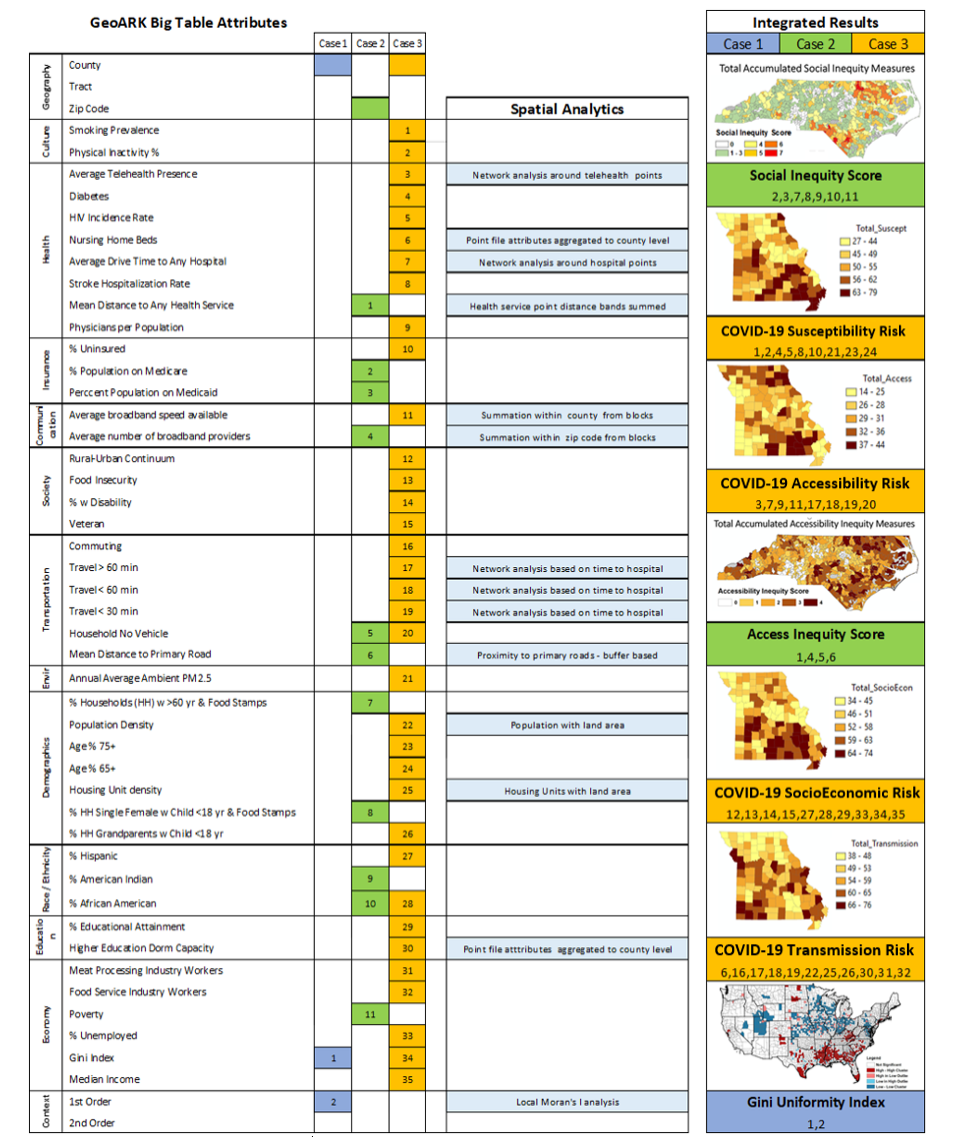
Case study examples of GeoARK attributes, processes, and outcomes. Each case study is given a color. Attributes used in their respective analyses are likewise color coded. Those attributes derived from spatial analytics are further noted. GeoARK: Geospatial Analytical Research Knowledgebase.

Results are presented as 3 case studies (Figure 3) utilizing the GeoARK system. The case studies cover (1) the development of new uniformity measures providing insight into health outcomes (blue), (2) telehealth program evaluation of both growth and impact on rural health access and equity (green), and (3) the development of COVID-19 risk factor assessments (orange). These examples demonstrate the GeoARK system’s utility and practical application in support of health research questions.

#### Case Study 1: Associations - Gini Index Example - Geography: County and State

Complex questions: How do health outcomes relate to geographic clustering of income inequality within the United States? Can a new spatial uniformity measure be created to aid understanding of income inequality?

This case study merged geospatial analytics with big data approaches to maximizing the use of the Behavioral Risk Factor Surveillance System (BRFSS) [55] and the ACS [56]. This study examined the income inequality hypothesis using cluster and outlier spatial analysis [57]. We applied this geospatial approach to create 3 innovative measures that captured uniformity in income inequality. We examined the ways that the Gini coefficient and 3 new spatial uniformity measures were associated with health outcomes. Specifically, the uniformity measures capture the extent to which (1) inequality is uniformly distributed spatially in states regardless of whether the level is high or low, (2) the extent to which states are more uniformly high in inequality across space, and (3) the extent to which they are more uniformly low in inequality. We conclude that residents of states that have more uniformly high inequality across space are more likely to report worse outcomes across several health measures. This case study showed that geospatial big data approaches can extend research on public health topics involving traditional survey data [58]. This also demonstrated how even 1 variable (in this case the Gini index), when spatially analyzed, can create new and useful insights into health investigations and their interpretation.

#### Case Study 2: Health Equity - Telemedicine Program Reach - Geography (Zip Code Tabulation Areas)

Complex questions: Who and where are the most vulnerable populations in terms of social inequity? Does the telehealth program address these vulnerable populations?

Utilizing aggregated telehealth use data, this case study evaluated a telehealth program’s reach, growth, and potential to address equity issues in rural areas. Significant inequities exist and can vary across scales from blocks to neighborhoods to regions [18]. From the occurrence data, the demand for receiving care via the program steadily increased over the 4 quarters, especially in rural areas. Three geospatially based health measures were created to assess and describe context: the social inequity score, the access inequity score, and a combined inequity score. In total, 11 measures, including social determinants (n=7, 64%) and access measures (n=4, 36%), were compiled from 5 sources and tabulated at the zip code level. GeoARK permits selection of both social elements and infrastructure-related accessibility elements. The social elements were pulled from multiple census sources, while the accessibility measures were created through geoanalytics and compiled into zip code boundaries for comparison. To assess the overall context of the delineated reach of the program, a mean combined inequity score was calculated for each zip code and for all zip codes. In zip codes where telemedicine encounters occurred, the population served had higher levels of social inequity and lower access in comparison to both state and rural levels. This telehealth program assessment of health inequity and access in rural regions demonstrated the program’s promising reach to vulnerable populations, as associated with the social and accessibility factors measured. These results supported maintaining and continued development of policies for affordable and on-demand telemedicine programs for providing care to rural populations facing inequities [26].

#### Case Study 3: Population Health - COVID-19 Risk - Geography (County)

Complex questions: What are the magnitudes of select risk factors, and where are they most prevalent in Missouri? What are the areas of compounded impacts, and do they cluster?

Studies of this COVID-19 pandemic require vast amounts of integrated data to create understanding that can then support decisions. We utilized GeoARK to extract and create 6 distinct thematic risk assessment databases for Missouri. The risk areas assessed included individual susceptibility or risk, potential transmission or community risk, socioeconomic contextual risk, accessibility constraints, health culture risk, and, finally, the exposure risk based on current case loads of COVID-19 at the county level. The goal of this project is to support data-driven decision-making processes across levels of government and health care providers to enable incorporation of significant risk factors associated with their specific populations and potential synergies and enable preparation for resilience and mitigation efforts across rural counties.

**Figure 4:**
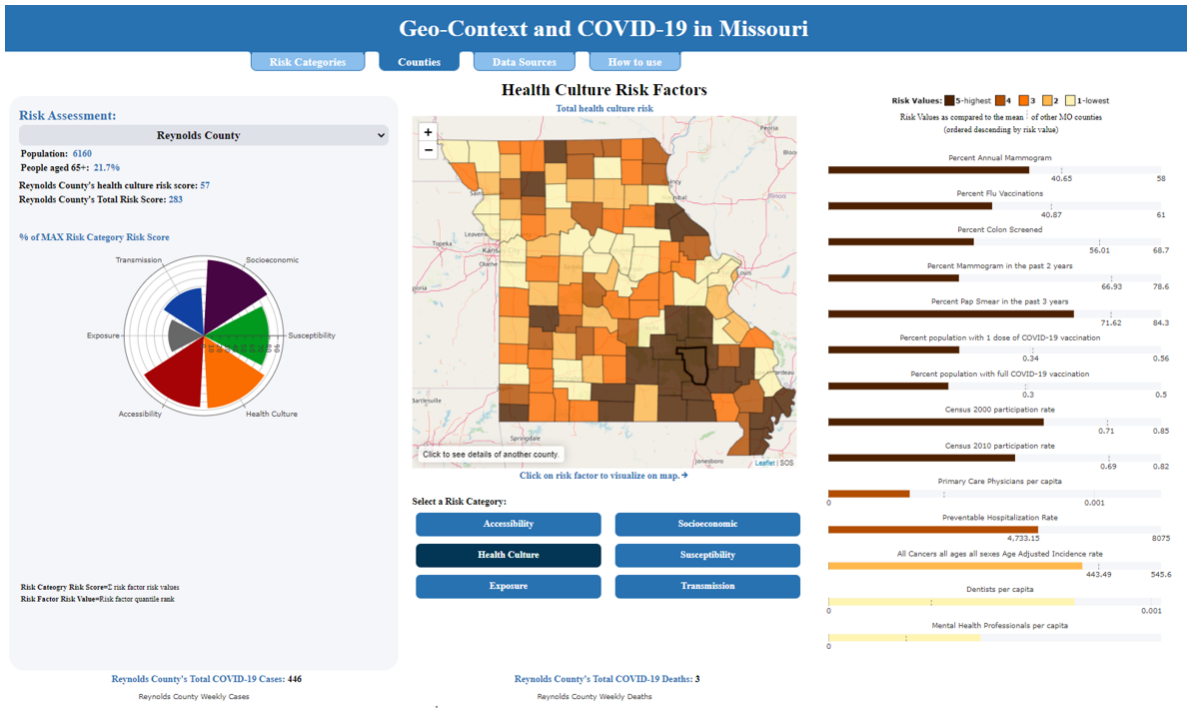
Screen capture of the "Geo-Context and COVID-19 in Missouri" dashboard interface populated with GeoARK parameters. GeoARK: Geospatial Analytical Research Knowledgebase.

The GeoARK data extraction and build for these risk databases included a selection of 325 (5.91%) elements from the current catalogue of 5500, integrating 35 different sources. A subset (total of 91 [28%] across all 6 areas) were then selected for use in the calculation of total risk scores for each assessment area. More specifically, components included known and possible comorbidities and age breaks; commuting, migration, worker types, group gatherings, and living situation; race, ethnicity, disability, insurance status, veteran status, and education level; development and inclusion of various hospital, nursing homes, and telehealth access measures; and broadband metrics. Ordinary least squares regression was used to evaluate combinations of explanatory variables. Selected variables within each risk category then had quintiles calculated to create comparative categorical groups for each risk variable, with higher values assigned to worse risk. Cumulative risk scores were assembled for each risk category, as well as an overall composite risk score. These values were then analyzed using Local Moran’s I, similarity analysis, and spatially constrained multivariate clustering to inform regional grouping outcomes. Through spatial analytics, differences in both the magnitude of risk and the substance of that risk, among and between rural and urban counties, were found. Missouri’s spatial diversity is evident in the variability of overall risk across the 6 factor areas developed as well as the 6 region-based groups of counties sharing similar risk traits. The results are queryable through the Geo-Context and COVID-19 website (Figure 4) [59].

These research results enhance the understanding of COVID-19 behavior and enable preparation for resilience in rural populations. It is important to understand the context and interrelationships of various risk factors occurring within the state in order to better understand the potential pathways for disease as well as what nuances in mitigation strategies are needed to address specific populations. There is no 1-size-fits-all solution for the diversity found through spatial analysis of risk. The ability to address issues that are most influencing the health of a particular region or population is paramount to equality in care.

## Discussion

### Principal Findings

There are unique innovations interwoven within the design of the GeoARK system. It has taken the multilayered world of typical GIS analysis and flattened it. The incorporation at each location of keys (ie, geographic-level Federal Information Processing Standard [FIPS] code, zip code) creates bridges for associated attribution to be incorporated into the GeoARK-BT. Other information is integrated through geospatial location, leveraging the fact that the information occupies the same location on the earth’s surface. For each point, the various scales, resolutions, information, and accuracies are captured as associated attributes of the particular data ingested. Through the integrated data services and analytical tools of this project, complex queries can be posed and associations explored. This is enabled only when spatial contexts have been quantified and thousands of factors associated spatially.

The enhanced analytics can provide a catalyst for health researchers to move beyond basic thematic mapping. In many, if not most, cases, the true benefit of using location is in the creation of new associations between data elements and subsequent creation of new information. The generation of this new quantified, tabular information is the real power of geospatial information and GeoARK.

### Benefits and Opportunities

The GeoARK system facilitates the use and integration of geoinformatics within the broad health-based user community. There is a high level of effort and expertise required to locate, compile, transform, standardize, and then integrate the multifaceted data required to adequately understand the context of eHealth events. It is critically important that health research be buoyed with access to the GeoARK system as it decreases duplication of effort, allows comparisons across a much broader set of potential variables, and extends the breadth and scope of investigations beyond the boundaries of conventional variable thematic mapping.

The linkage of results to a specific geographic scale, and the concurrent interpretation of them in context, is a growing requirement of sociological and health research. Because the GeoARK system has precalculated and captured the distance-based relationships of neighbors, features, and other spatial context, the project will aid researchers in development of comparable populations at varying scales. This could be within a certain aspect of interest (rural-urban) or geography (county, zip code, or tract).

A focus of potential benefit will be the use of the GeoARK system in research design. Meaningful health analytics typically address developing and testing hypotheses to contrast and compare 1 group (reference) to another (comparison). The ability to “know” and possibly choose to control for “outside” variables (eg, environmental, social, cultural, infrastructure, or other factors) during the design of a study or trial may provide a clearer picture of the health aspect under investigation. The ability to tighten the research question or clinical trial, and its reference groups, leads to higher potential to achieve significant insights.

### Challenges and Limitations

The patient protections provided through the implementation and interpretation of the Health Insurance Portability and Accountability Act of 1996 (HIPAA) impacts the geographic scales at which we can investigate the detailed distributions of disease and health effects. Although all disease occurs as events, the way in which aggregation, compilation, and subsequent roll-up of these events into geographies has a dampening effect on most, if not all, attempts to drill deeper into the spatial context and phenomenology of diseases.

Variability and uncertainty exist within all data collected by organizations as it was in the pursuit of a mandated purpose. Biases, ethical issues, and errors complicate the systematic integration of heterogeneous information into any database. By using location, it is hoped that these biases and other issues will be more clearly brought to light.

The modifiable areal unit problem has the potential to create problems with representation of certain types of data. By assembling these data across a variety of raster resolutions, the scales of representation can be tested and understood so that use of these data at any scale would be accompanied by a “fitness of use” measure that can be presented to the user. Because a range of geographies is captured upon integration within the design of GeoARK, these comparisons can also be tested for stability and significance across a range of scales. This allows researchers to evaluate at what level the component of interest manifests itself and therefore permit identification of the proper level for intervention (as well as what determinants are amenable to this process) or information that can be used for avoidance of a particular type of disparity in a particular area.

The evolution of the GeoARK-BT to the fully envisioned system as a web-based portal with robust data and research services has many hurdles to overcome. These include data usage agreements, compute scaling, cloud service strategy, data-as-a-service management strategy, security and compliance adjustments, performance tuning, build out of analytics, and cost constraints.

However, the biggest challenge remaining for health researchers is to learn to think spatially about their problems and broaden their research questions into the multifactor, multiscale arenas of investigation that the GeoARK system supports.

### Conclusion

This paper describes and outlines the design, compilation, and assembly of the GeoARK system, a spatially referenced data table that facilitates the integration and standardization of sociocultural, infrastructural, environmental, and health-related data into a common, extractable, and analytical framework.

The GeoARK system provides the ability to identify, mitigate, and contextualize health disparities. It provides health researchers with an integrated big data repository that can be searched to enable stronger research designs, for example, develop sampling/surveillance approaches or clinical trial focus. Using context across a broad range of data, research topics surrounding avoidance, fairness, equity, justice, and acceptability within, or for, a given location can be pursued. GeoARK supports user-based query, contextual analysis, and visualization to investigate relationships among the integrated data layers as well as discover patterns of interest for health research.

There are myriad ways that the GeoARK system, as a service, can be used in future analyses in order to better understand health disparities and other research issues. This system enables researchers to draw deeper and more broadly applicable empirical evidence for health research and associated outcomes, as well as supporting AI/ML-friendly data extracts that can then leverage new spatial associations.

This framework provides benefit to eHealth-related research, applications, and policy evaluation by the broader health community and has the potential to transform health research from a layer-based mentality to an interactive integrated contextual knowledge platform.

## Appendix

Multimedia Appendix 1 Supplementary File providing 3 pages of both the tabular data source listing and the geospatial source listing of files integrated or being integrated into the GeoARK-BT and available through the GeoARK system for support of complex queries and data selection in support of the health research community. GeoARK: Geospatial Analytical Research Knowledgebase; GeoARK-BT: GeoARK-Big Table.

## Abbreviations

ACS: American Community Survey
AI: artificial intelligence
FIPS: Federal Information Processing Standard
GeoARK: Geospatial Analytical Research Knowledgebase
GeoARK-BT: GeoARK-Big Table
GIS: Geographic Information System
ML: machine learning
NIH: National Institutes of Health

## Data Availability

The data underlying this paper will be shared through the Geospatial Analytical Research Knowledgebase (GeoARK) service. Questions can be sent to the corresponding author.
