# Supplementary material for: Investigating Health Context Using a Spatial Data Analytical Tool: Development of a Geospatial Big Data Ecosystem
Source: JMIR Med Inform. 2022 Apr 6;10(4):e35073. doi: 10.2196/35073 (PMC9021952; doi:10.2196/35073)
Supplement: Multimedia Appendix 1 [file medinform_v10i4e35073_app1.pdf]

Supplementary File A

This Supplementary File provides over the next 3 pages both the tabular data source listing and the geospatial source listing of files integrated into the GeoARK Big Table and available through the GeoARK System for support of complex queries and data selection in support of the health research community.

GeoARK's Tabular Datasets

The listing below provides the tabular data set name, the number of attributes from that file, the files native format, and the associated URL link for that dataset. This listing shows by example that multiple tabular data sources can and have been linked and integrated into GeoARK.

	Dataset Name	NumAttrib	Format	Source URL
1	GeoARK Base	21	csv	NA
2	USA Facts Covid-19 Cases	577	csv	https://usafactsstatic.blob.core.windows.net/public/data/covid-19/
3	USA Facts Covid-19 Deaths	573	csv	https://usafactsstatic.blob.core.windows.net/public/data/covid-19/
4	Harvard Global Health Institute COVID-19 Hospital Capacity Estimates	111	xlsx	https://globalepidemics.org/hospital-capacity/
5	Food Access Research Atlas	145	xlsx	https://www.ers.usda.gov/webdocs/DataFiles/
6	Food Environment Atlas	277	xls	https://www.ers.usda.gov/webdocs/DataFiles/
7	Atlas of Rural and Small-Town America	232	xlsx	https://www.ers.usda.gov/webdocs/DataFiles/
8	Interactive Atlas of Heart Disease and Stroke	23	xlsx	https://www.cdc.gov/dhdsp/maps/atlas/index.htm#
9	Selected Economic Characteristics (DP03)	548	csv	https://data.census.gov/cedsci/table?tid=ACSDP5Y2018.DP03
10	Veteran Status (S2101)	480	csv	https://data.census.gov/cedsci/table?tid=ACSST5Y2018.S2101
11	Type of insurance; Medicare, Medicaid, VA, Public, private (S2704)	174	csv	https://data.census.gov/cedsci/table?tid=ACSST5Y2018.S2704
12	Housing details; vehicles avail; telephone service; occupants/room (DP04)	572	csv	https://data.census.gov/cedsci/table?tid=ACSDP5Y2018.DP04
13	Race & Hispanic - Ethnicity (DP05)	356	csv	https://data.census.gov/cedsci/table?tid=ACSDP5Y2018.DP05
14	Smoking Prevalence 1996-2012 (Behavioral Risk Factor Surveillance System)	55	xlsx	https://www.cdc.gov/brfss/
15	Community Characteristics by Sex (S0801)	74	csv	https://data.census.gov/cedsci/table?tid=ACSST5Y2017.S0801
16	Food Stamps/Supplemental Nutrition Assistance Program (SNAP) (S2201)	456	csv	https://data.census.gov/cedsci/table?tid=ACSST5Y2018.S2201
17	Population 60 years and over in the United States (S0102)	424	csv	https://data.census.gov/cedsci/table?tid=ACSST5Y2018.S0102
18	Selected Social Characteristics in the United States (DP02)	495	csv	https://data.census.gov/cedsci/table?tid=ACSDP5Y2018.DP02
19	EJSCREEN: Environmental Justice Screening and Mapping Tool	80	csv	https://www.epa.gov/ejscreen/

Supplementary File A - continued

GeoARK's Geospatial Datasets

The listing below provides the feature layer descriptive name and the associated Source for that dataset. This listing shows by example the breadth of spatial data sources that can and have been linked and integrated into GeoARK.

	Feature Layer Name	Source
1	National, State, County, Regional, and Local Parks or Forests	U.S. Geological Survey (USGS) 2018, Protected Areas Database of the United States (PAD-US)
2	Impervious Surface	Multi-Resolution Land Characteristics Consortium https://www.mrlc.gov/
3	Land Cover Change Index	Multi-Resolution Land Characteristics Consortium https://www.mrlc.gov/
4	Land Cover	Multi-Resolution Land Characteristics Consortium https://www.mrlc.gov/
5	Forest_Theme_Disturbance	Multi-Resolution Land Characteristics Consortium https://www.mrlc.gov/
6	Cropland Data Layer	USDA, National Agricultural Statistics Service: CropScape https://nassgeodata.gmu.edu/CropScape
7	Corn Frequency	USDA, National Agricultural Statistics Service: CropScape https://nassgeodata.gmu.edu/CropScape
8	Soybean Frequency	USDA, National Agricultural Statistics Service: CropScape https://nassgeodata.gmu.edu/CropScape
9	Cotton Frequency	USDA, National Agricultural Statistics Service: CropScape https://nassgeodata.gmu.edu/CropScape
10	Wheat Frequency	USDA, National Agricultural Statistics Service: CropScape https://nassgeodata.gmu.edu/CropScape
11	EPA Comprehensive Environmental Response Compensation and Liability Information System	United States Environmental Protection Agency (EPA)
12	EPA Emergency Response (ER) Risk Management Plan (RMP)	United States Environmental Protection Agency (EPA)
13	EPA Emergency Response (ER) Toxic Release Inventory (TRI)	United States Environmental Protection Agency (EPA)
14	EPA Emergency Response (ER) Toxic Substances Control Act (TSCA)	United States Environmental Protection Agency (EPA)
15	EPA Resource Conservation and Recovery Act Treatment Storage and Disposal (RCRATSD)	United States Environmental Protection Agency (EPA)
16	Solid Waste Landfill Facilities	Oak Ridge National Laboratory (ORNL)
17	Child Care Centers	Oak Ridge National Laboratory (ORNL)
18	Colleges and Universities Campuses	Oak Ridge National Laboratory (ORNL)
19	Private Schools	Oak Ridge National Laboratory (ORNL)
20	Public Schools	Oak Ridge National Laboratory (ORNL)
21	Supplemental Colleges	Oak Ridge National Laboratory (ORNL)
22	American Red Cross Chapter Facilities	TechniGraphics, Inc (TGS); American Red Cross
23	Emergency Medical Service (EMS) Stations	TechniGraphics, Inc (TGS)
24	Fire Stations	U.S. Geological Survey (USGS)
25	Local Emergency Operations Centers (EOC)	TechniGraphics, Inc (TGS)
26	National Shelter System Facilities	FEMA GIS
27	PSAP 911 Service Area Boundaries	TechniGraphics, Inc (TGS)
28	Biodiesel Plants	Oak Ridge National Laboratory (ORNL)
29	Bottom Wells	Oak Ridge National Laboratory (ORNL)
30	Electric Planning Areas	Oak Ridge National Laboratory (ORNL)
31	Electric Power Transmission Lines	Oak Ridge National Laboratory (ORNL)
32	Electric Retail Service Territories	Oak Ridge National Laboratory (ORNL)
33	Electric Substations	Oak Ridge National Laboratory (ORNL)
34	Environmental Protection Agency (EPA) Facility Registry Service (FRS) Power Plants	United States Environmental Protection Agency (EPA)
35	Ethanol Plants	Oak Ridge National Laboratory (ORNL)
36	Ethanol Transloading Facilities	Oak Ridge National Laboratory (ORNL)
37	Hydrocarbon Gas Liquid Pipelines	U.S. Energy Information Administration (EIA)
38	Independent System Operators	Oak Ridge National Laboratory (ORNL)
39	Liquified Natural Gas Import Exports and Terminals	Oak Ridge National Laboratory (ORNL)
40	Natural Gas Compressor Stations	Oak Ridge National Laboratory (ORNL)
41	Natural Gas Import and Export	Oak Ridge National Laboratory (ORNL)
42	Natural Gas Pipelines	U.S. Energy Information Administration (EIA)
43	Natural Gas Processing Plants	Oak Ridge National Laboratory (ORNL)
44	Natural Gas Receipt Delivery Points	Oak Ridge National Laboratory (ORNL)
45	Natural Gas Storage Facilities	Oak Ridge National Laboratory (ORNL)
46	Oil and Natural Gas Fields	Oak Ridge National Laboratory (ORNL)
47	Oil and Natural Gas Interconnects	Oak Ridge National Laboratory (ORNL)
48	Oil and Natural Gas Platforms	Oak Ridge National Laboratory (ORNL)
49	Oil and Natural Gas Wells	Oak Ridge National Laboratory (ORNL)
50	Oil Refineries	Oak Ridge National Laboratory (ORNL)
51	Oil Refinery (Polygon)	Oak Ridge National Laboratory (ORNL)
52	Petroleum Ports	Oak Ridge National Laboratory (ORNL)
53	Petroleum Terminals	Oak Ridge National Laboratory (ORNL)
54	POL Pumping Stations	Oak Ridge National Laboratory (ORNL)
55	Power Plants	Oak Ridge National Laboratory (ORNL)
56	Public Refrigerated Warehouses	International Association of Refrigerated Warehouses
57	DoD Sites Boundaries (Public)	Source compiled by the Defense Installation Spatial Data Infrastructure (DISDI) Program
58	DoD Sites Points (Public)	Source compiled by the Defense Installation Spatial Data Infrastructure (DISDI) Program
59	Formerly Used Defense Sites (FUDS) Projects (Points)	United States Army Corps of Engineers (USACE)
60	Formerly Used Defense Sites (FUDS) Public Munitions Response Sites (MRS)	United States Army Corps of Engineers (USACE)
61	Formerly Used Defense Sites (FUDS) Public Properties	United States Army Corps of Engineers (USACE)
62	Formerly Used Defense Sites (FUDS) Public Property Boundaries	United States Army Corps of Engineers (USACE)
63	Interim Risk Management (IRM) Project Boundary	United States Army Corps of Engineers (USACE)
64	Interim Risk Management (IRM) Property Point	United States Army Corps of Engineers (USACE)
65	LandScan USA	Oak Ridge National Laboratory (ORNL)
66	Local Law Enforcement Locations	ORNL, National Geospatial-Intelligence Agency (NGA) Homeland Security Infrastructure Program (HSIP)
67	Prison Boundaries	Oak Ridge National Laboratory (ORNL)
68	Agricultural Minerals Operations	U.S. Geological Survey (USGS)
69	Construction Minerals Operations	U.S. Geological Survey (USGS)
70	Crushed Stone Operations	U.S. Geological Survey (USGS)
71	Ferrous Metal Mines	U.S. Geological Survey (USGS)
72	Ferrous Metal Processing Plants	U.S. Geological Survey (USGS)
73	Mines and Mineral Resources	TechniGraphics, Inc (TGS)
74	Nonferrous Metal Mines	U.S. Geological Survey (USGS)
75	Nonferrous Metal Processing Plants	U.S. Geological Survey (USGS)
76	Refractory Abrasive and Other Industrial Mineral Operations	U.S. Geological Survey (USGS)
77	Sand and Gravel Operations	U.S. Geological Survey (USGS)
78	Uranium and Vanadium Deposits	U.S. Geological Survey (USGS)
79	US Coal Fields	U.S. Geological Survey (USGS)
80	National Flood Hazard Layer (NFHL)	Federal Emergency Management Agency (FEMA)
81	Fire Perimeters	National Interagency Fire Center (NIFC)
82	Historical Fire Perimeters	National Interagency Fire Center (NIFC)
83	Historical Holocene Volcano Locations	U.S. Geological Survey (USGS) National Atlas of the United States
84	Historical Significant Volcanic Eruption Locations	National Oceanic and Atmospheric Administration (NOAA), National Geophysical Data Center
85	Historical Tornado Tracks	National Oceanic and Atmospheric Administration (NOAA), National Weather Center
86	Historical Tropical Storm Tracks	National Oceanic and Atmospheric Administration (NOAA), National Hurricane Center
87	Landslide Regions	U.S. Geological Survey (USGS)
88	Plate Boundaries	U.S. Geological Survey (USGS)

89	Seismic Ground Motion Hazards with 10 Percent Probability	U.S. Geological Survey (USGS) Earth Science Information Center
90	Seismic Ground Motion Hazards with 2 Percent Probability	U.S. Geological Survey (USGS) Earth Science Information Center
91	Hospitals	Oak Ridge National Laboratory (ORNL)
92	Nursing Homes	Oak Ridge National Laboratory (ORNL)
93	Public Health Departments	TechniGraphics, Inc (TGS)
94	Urgent Care Facilities	TechniGraphics, Inc (TGS)
95	Veterans Health Administration Medical Facilities	TechniGraphics, Inc (TGS)
96	All Places of Worship	HIFLD
97	Cruise Line Terminals	Department of Homeland Security (DHS) (iMap data)
98	Major Sport Venues	Oak Ridge National Laboratory (ORNL)
99	Major Sport Venues Usage	Oak Ridge National Laboratory (ORNL)
100	Mobile Home Parks	Oak Ridge National Laboratory (ORNL)
101	Public Transit Routes	Oak Ridge National Laboratory (ORNL)
102	Public Transit Stations	Oak Ridge National Laboratory (ORNL)
103	Rail Company	Oak Ridge National Laboratory (ORNL)
104	Road Tunnels	TechniGraphics, Inc (TGS)
105	Stations and Transfers	Oak Ridge National Laboratory (ORNL)
106	Trails	USGS, NGTOC, NGA
107	National Hydrography Dataset (NHD) Waterbodies - Small Scale	U.S. Geological Survey (USGS)
